# Study of bearing strength for injection molded GFRPP composites under dry and wet conditions

**DOI:** 10.1038/s41598-022-21539-z

**Published:** 2022-10-20

**Authors:** A. A. Megahed, M. M. Osama, A. I. Selmy, Ayman M. M. Abdelhaleem

**Affiliations:** 1 Mechanical Design and Production Engineering Department, Faculty of Engineering, Zagazig University, P.O. Box: 44519, Zagazig, Al-Sharqia, Egypt; 2 Mechanical Engineering Department, Higher Technological Institute, Tenth of Ramadan City, Al-Sharqia, Egypt

**Keywords:** Engineering, Materials science

## Abstract

Thermoplastics and fiber-reinforced thermoplastics represent great deals in nowadays industries and applications where some of these applications are projected to wet environment. The present study investigates the effect of water moisture on the bearing strength (BS) of Polypropylene (PP) and glass fiber (GF) reinforced Polypropylene (GFRPP) composites. PP and GFRPP are produced by injection molding using different GF weight fractions (wt%), 10, 20, and 30 wt%, and two different initial fiber lengths 12 and 24 mm. A burnout test indicated that produced specimens with 12 mm long fibers have higher final fiber lengths than those made of 24 mm long fibers. More water was absorbed for higher GF weight fractions. The results of the dry bearing test showed higher bearing strengths for specimens with higher GF wt% and longer fibers. The same observation was obtained from wet tests, while, wet-tested specimens of all compositions have higher strengths than their dry counterparts. Strain-at-break seemed to be significantly reduced by water absorption for all specimens. Specimens tested in wet conditions have different fracture morphology than dry ones due to the change in the mechanical behavior of the materials after water immersion.

## Introduction

Thermoplastics and their composites have a huge importance in several engineering fields according to their unique characteristics. Recyclability, relatively low weights, nontoxicity, and cost-effectiveness are major advantages of these materials that make them one of the best candidates in aircraft, medical and marine applications. As they have several marine applications, they are directly subjected to water which may ingress into the material causing a change in the material's mechanical characteristics^1^. The water uptake and its effect on mechanical properties and tribological properties of the polymeric matrix composites have been discussed in numerous studies. Guo and Kethineni^2^ found that the tensile strength of injection molded PP reinforced by 20% GF composite decreased insignificantly after water immersion for 24 h. Also, less water sorption for GF reinforced/PP than pure PP was observed. The same results were obtained by Guo et al.^3^ on High-density Polyethylene (HDPE) after water immersion for 48 h. while a slight decrease occurred for HDPE/Carbon fibers (CF) due to interfacial bond weakening between the matrix and the fibers as a result of water diffusion. More water absorption was observed as CF percentages increased. Deng et al.^4^ noticed that glass mat (GMT) reinforced PP absorbed water more than Isotropic PP. A negligible reduction in tensile strength and Young’s modulus as a result of water absorption was noticed for PP and GMT composite laminates.

Arif et al.^5^ studied a twin-screw-extruded and injection molded Polyamide 66 (PA66) / 30 wt% short glass fiber composite under 0%, 50%, and 100% relative humidity (RH). Stiffness and flexural strength of the composite decreased with increased RH due to damage and plasticization effect while strain to failure was higher on higher RHs. Carrascal et al.^6^ hinted that as the fiber content of Polyamide (PA)/GF increased the water absorption decreased, while the tensile strength decreased as humidity increased. Bergeret et al.^7^ in their study of water sorption on 30 wt% GF reinforced thermoplastics (PA66, Polybutylene terephthalate (PBT), and Polyethylene terephthalate (PET) ) under high temperatures (90–135 °C) found a significant drop in mechanical properties (tensile and Impact strengths). Bergeret et al.^8^ obtained ultimate tensile and impact strengths on oxygen-free-water-immersed short GF reinforced thermoplastics (PET + 45 wt% GF, PA66 + 30 wt% GF, and PBT + 15 wt% GF) under different aging conditions (Pressure and Temperature). 50–90% reduction in ultimate stress of PET, PA66, and PBT composites respectively after 50–200 h while a slight increase in impact strength of PET was noticed at early times of aging.

Several studies have been made on the influence of GF addition to PP/Natural fiber composites where a noticeable decrease in water absorption was noticed as GF was introduced to those composites at room temperature^9–16^. Also, a reduction in strength properties was observed after water sorption^15^ and long-term water aging^13,17^. Thwe and Liao^13^ noticed a decrease in flexural strength and stiffness for Bamboo fiber/PP and Bamboo fibers/GF/PP composites after water aging at 25–75 °C depending on the time and temperature of aging. While Mohebby et al.^18^ concluded that water absorption of hybrid wood-flour/GF reinforced PP composites elevated as more GF was introduced to the composite without the coupling agent. When the coupling agent was applied to the composite this observation was opposed. Shakeri and Raghimi^19^ observed a decrease in water absorption as GF content increased for GF Recycled-Newspaper PP hybrid composite.

Materials inevitably weakened at joining points^20^ which require holes as bolts are considered to be a major joining technique^20–26^. Water uptake by the material could affect material bearing strength and this effect should be studied where it is a good representation of joint strength under a wet environment. According to our literature review, there is no systematic study has been made on the effect of water absorption on the bearing strength of GF reinforced thermoplastic composites. Moreover, previous studies were restricted mostly to the effect on tensile, flexural, and impact strengths. The current work will focus on studying the bearing strength at dry and wet conditions for PP and GFRPP composites with different GF weight fractions.

## Materials and experimental work

### Materials

The matrix was made of Copolymer polypropylene (PP) pellets (413MNK45) supplied by SABIC®—Egypt and made especially for injection molding with a density of 0.905 g/cm^3^. A melt flow rate of PP was 70 g/10 min at 230 °C and 2.16 kg. 13 µm diameter E-glass chopped strands (GF) supplied by JUSHI® were used as reinforcements with 2.55 g/cm^3^ density. Two chop lengths were used for GF, 12 and 24 mm.

### Composite preparation

**Figure 1:**
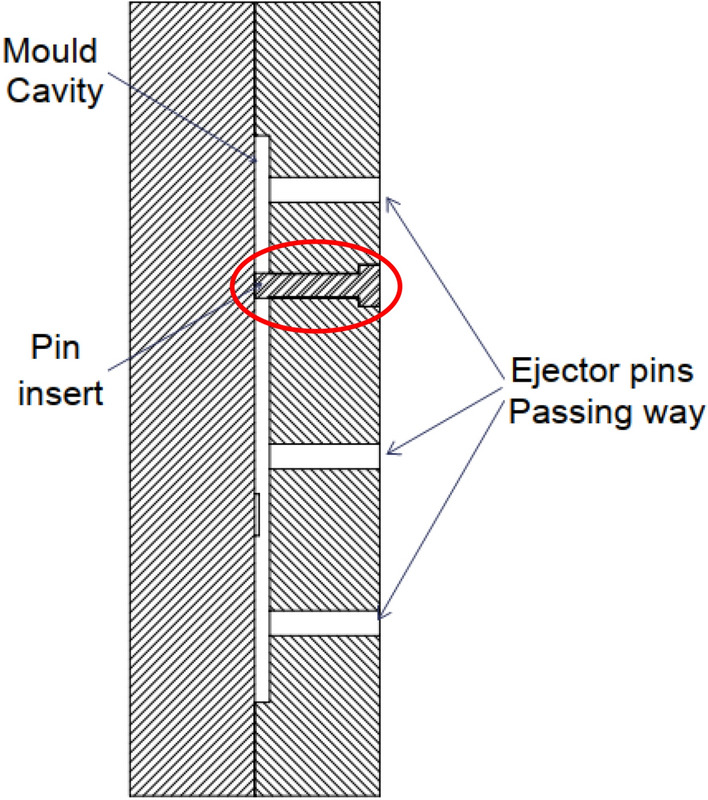
Bearing mold.

Bearing test specimens were manufactured using HAITIAN PL1200 injection molding machine with a maximum clamping force of 1200 kN, the injection parameters are shown in Table 1. The mold was designed, manufactured, and examined several times to check its suitability for producing test specimens. In this mold, the same direction of the flow for the plastic is considered for each specimen to avoid the probability of weld lines formation which prevents cracks creation. A core pin was inserted inside the mold cavity to produce a specimen with a ready Φ6 mm hole (Fig. 1).

**Table 1:** Injection parameters.

Parameter	Stages				
	1	2	3	4	5
Barrel temperature (°C)	140	160	180	220	224
Injection speed^a^	8%	10%	12%	8%	10%
Injection pressure (bar)	55	55	55	60	60
Charging pressure (bar)	100	100	100	–	–
Back pressure (bar)	3	3	3	–	–

^a^Injection speed percentage of the maximum injection speed.

**Figure 2:**
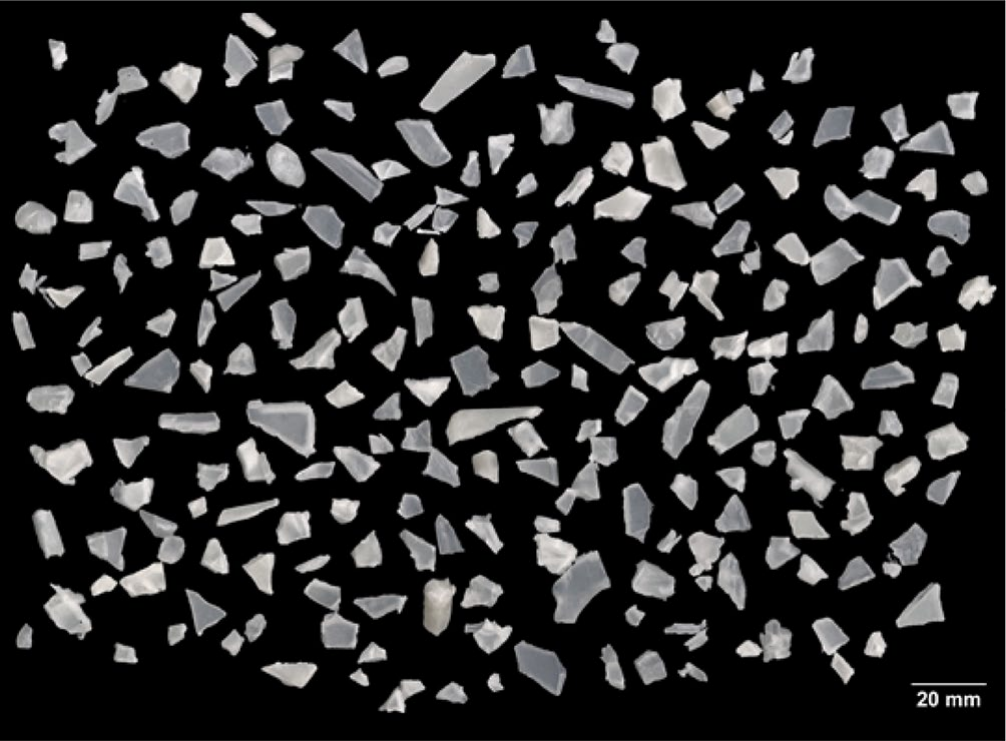
The granules after the crushing process.

The manufacturing process was conducted as follows; neat PP bearing specimens were injected firstly then PP pellets were mechanically blended with GF with different weight fractions of 10, 20, and 30 wt% and different feedstocks 12 mm and 24 mm. The mixing process was performed in the hopper of the injection molding machine in the solid state where no pre-manufactured GFRPP pellets were used. The mixture was first fed to the extruder of the injection molding machine to produce pre-samples. Pre-samples with their sprue and runners were crushed in a crusher forming small granules, the specifications of the crusher are tabulated in Table 2. The granules sizes were captured using an optical scanner as shown in Fig. 2 and then analyzed using Fiji ImageJ application. The average size of the small granules was found to be 66.3 mm^2^. The small particles were re-injected once again to obtain the final test specimens. The main purpose of these stages is to gain a better distribution of the GF into the PP^27,28^. The same injection molding conditions and parameters were used for the two injection molding processes. The codes and designations of specimens manufactured for dry and wet bearing tests are illustrated in Table 3.

**Table 2:** The crusher specifications.

Parameter	Value
Crusher type	Shrouded
Number of knives	3 pairs
Screen hole size	8 mm
Rotational grinding speed of knives	460 R.P.M

**Table 3:** Specimens’ codes.

Specimen code		PP, wt%	GF, wt%	Feedstock Fiber length (mm)
Dry condition	Wet condition			
DB00	WB00	100	0	–
DB1012	WB1012	90	10	12
DB2012	WB2012	80	20	
DB3012	WB3012	70	30	
DB1024	WB1024	90	10	24
DB2024	WB2024	80	20	
DB3024	WB3024	70	30	

### Water uptake

A group of bearing test specimens were weighted and then immersed for 100 days in distilled water at room temperature according to ASTM D5229/D5229M. Before immersion, the specimens were kept in a dry condition away from humid climates. Surface moisture was carefully removed after the specimens were picked up from the water to be weighed periodically to examine the material water sorption. Surface moisture was removed using clean, dry, and dustless tissues. An accurate four-digit balance (Mettler AE200), with 0.0001 g resolution, was used in the weighting process. The percentage of water absorbed (WS%) was calculated for each specimen according to the following equation^28–30^:

1 $$WS\%= \left(\frac{{W}_{t}-{W}_{0}}{{W}_{0}}\right)*100$$

where, ${W}_{0}$ is the initial specimen weight and ${W}_{t}$ is the specimen weight after a period (t). Introducing materials to a wet environment or immersing them in water leads to water sorption by material and the percentages of water sorption vary between different materials. Obeying Fickian diffusion law waters were assumed to penetrate inside materials where the mass of absorbed water increases with the square root of time in a linear manner. According to the type of material, the water absorption slows down until saturation is reached where approximately no more water penetrates the material. The Fickian model express’s diffusion of water using the following equation;

2 $$D=\pi {\left(\frac{h \left({M}_{2}-{M}_{1}\right)}{4{M}_{\infty }(\sqrt{{t}_{2}}-\sqrt{{t}_{1}})}\right)}^{2}$$

where *D* is the coefficient of diffusion, ${M}_{\infty }$ is the weight of the water absorbed at saturation, $h$ is the thickness of the specimen,${M}_{1}$ and ${M}_{2}$ are the moisture contents at times ${t}_{1}$ and ${t}_{2}$, respectively. The chosen times could be selected at the linear stages of water sorption.

### FTIR observation

Fourier transform infrared spectroscopy (FTIR) was performed to study the changes in the chemical composition of PP and GFRPP after being immersed in distilled water. These observations were to determine the potential degradation of neat PP and the PP matrix, fibers and interface of the GFRPP as an effect of water absorption. The aim was to correlate these observations with possible changes in bearing strength, bearing strain and chemical composition of PP and GFRPP after immersion. The test was also made on dried specimens after water immersion to investigate whether the effect of water immersion is temporary or permanent.

### Bearing strength test

**Figure 3:**
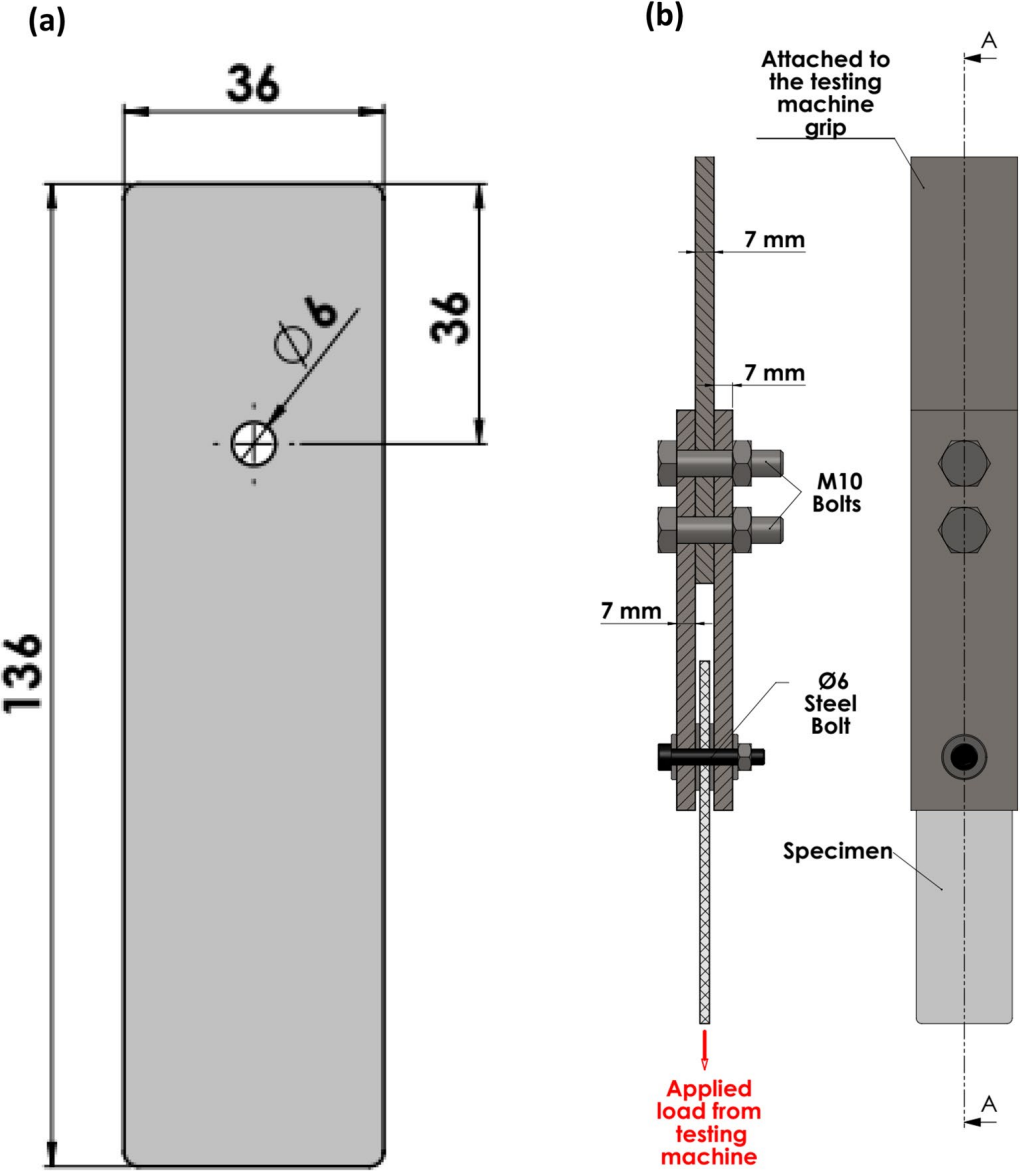
Bearing test; (**a**) specimen and (**b**) fixture.

A series of pin-bearing ASTM D5961 tests were conducted on both dry and wet conditions for all specimens using a universal testing machine (Testometric 200 kN) at room temperature. Standard test specimens were used to obtain bearing failure mode rather than net tension or shear-out modes that had lower loads associated with catastrophic fracture as recommended by previous studies^31,32^. The dimensions of the standard test specimen are illustrated in Fig. 3a where w/d = 6 and e/d = 6. The test fixture was manufactured from steel according to the geometry illustrated in Fig. 3b. Wet bearing tests were conducted immediately after specimens were picked up from water and surface moisture removal.

### Measurement of fiber length

**Figure 4:**
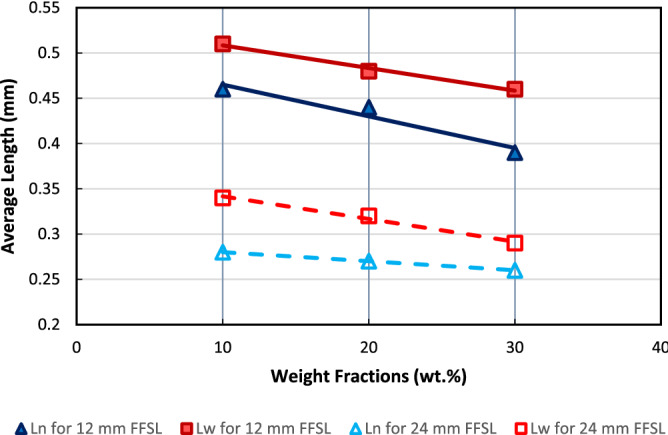
Weighted (*L*_*w*_) and number (*L*_*n*_) average lengths after burn-out of composites with different fiber feedstock lengths (FFSLs).

Fiber’s length measurements were conducted after complete matrix burn-out at 570 °C for 4 h on a muffle furnace leaving only fibers behind. The burn-out process was conducted for both FFSL (Fiber Feedstock lengths) of 12 and 24 mm. Then fibers were captured using optical scanners and analyzed using Fiji ImageJ application to determine the fiber lengths. As previous studies^33,34^ proposed, number average and weighted average lengths were used to indicate the average fiber lengths in the composite. The relations are expressed as follows;

3 $$L_{n} = \frac{{\sum F_{i} L_{i} }}{{\sum F_{i} }}$$

and

4 $$L_{w} = \frac{{\sum F_{i} L_{i}^{2} }}{{\sum F_{i} L_{i} }}$$

where ${ }L_{i}$ is the length of the fiber *i* in the sample and ${F}_{i}$ is the frequency of fiber length ${L}_{i}$. The weighted average fiber length ${L}_{w}$ is affected by the presence of longer fibers. While the number average fiber length ${L}_{n}$ is strongly affected by the fibers and fragments repetition. Mostly, ${L}_{n}$ is smaller than ${L}_{w}$^33,34^. Figure 4 represents both weighted and number average lengths of GF after the burn-out process.

## Results and discussion

### Weighted average and number-average fiber lengths

The results of this analysis, in Fig. 4, proved clearly that the fiber lengths decreased dramatically after the injection molding process^33–41^. The friction of the composite material with both the barrel and the screw generates dragging flow that produces a compound series of compressive forces, shearing, and differential motion of liquid polymer to the solid counterpart during the solid to melt transition. The combined effect causes severe damage to GFs^42^.

The reduction in the average fiber length of the glass fibers occurs in three stages. The first stage was through the first injection process as the screw extruder severely damages the fiber during the injection process resulting in a huge reduction in average fiber length^37^. The second stage was the crushing process which also plays a big role in reducing the lengths of the fibers along with composite smashing. The final stage happened in the second injection process while the smashed composite crumbs face the screw extruder once again^40,41^.

It is also noticed from Fig. 4 that, as fiber weight fraction increases a slight decrease in both weighted and number average fiber lengths is observed. This result is obtained also by Refs.^34–38,42^. The weighted and number average fiber lengths decrease with increased FFSL (Fiber Feedstock Lengths). The severe damage to the fiber lengths is majorly linked to high interaction that occurred between fibers at higher percentages of the fibers in the composite as concluded by Kumar et al.^34^. They reported that, when FFSL increases, both ${L}_{n}$ and ${L}_{w}$ increase for FFSL up to 9 mm, while for FFSL more than 9 mm both ${L}_{n}$ and ${L}_{w}$ decrease. More details including histograms for each weight fraction and FFSL was provided in previous study^43^. Throughout this work, based on the above results, FFSL of 12 mm and 24 mm will be referred to as “long fiber/Polypropylene (LFRPP)” and “short fiber/Polypropylene (SFRPP)”, respectively.

### Water absorption

**Figure 5:**
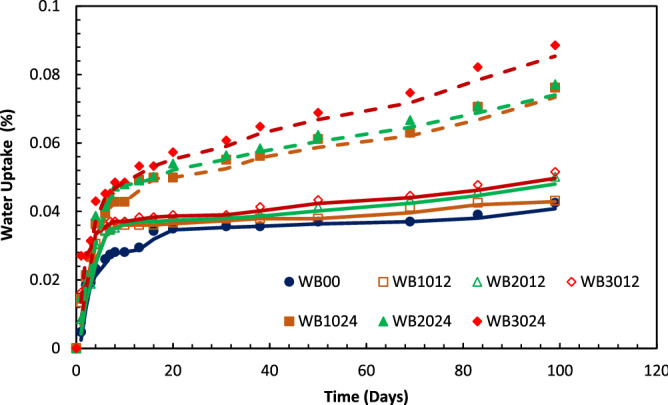
Water absorption of PP and GFRPP with different fiber lengths.

The water uptake of PP and GFRPP specimens with different fiber lengths was calculated as described in “Water uptake” from time to time and then plotted as shown in Fig. 5. Where the relation between water uptakes of the different specimens could be observed.

**Figure 6:**
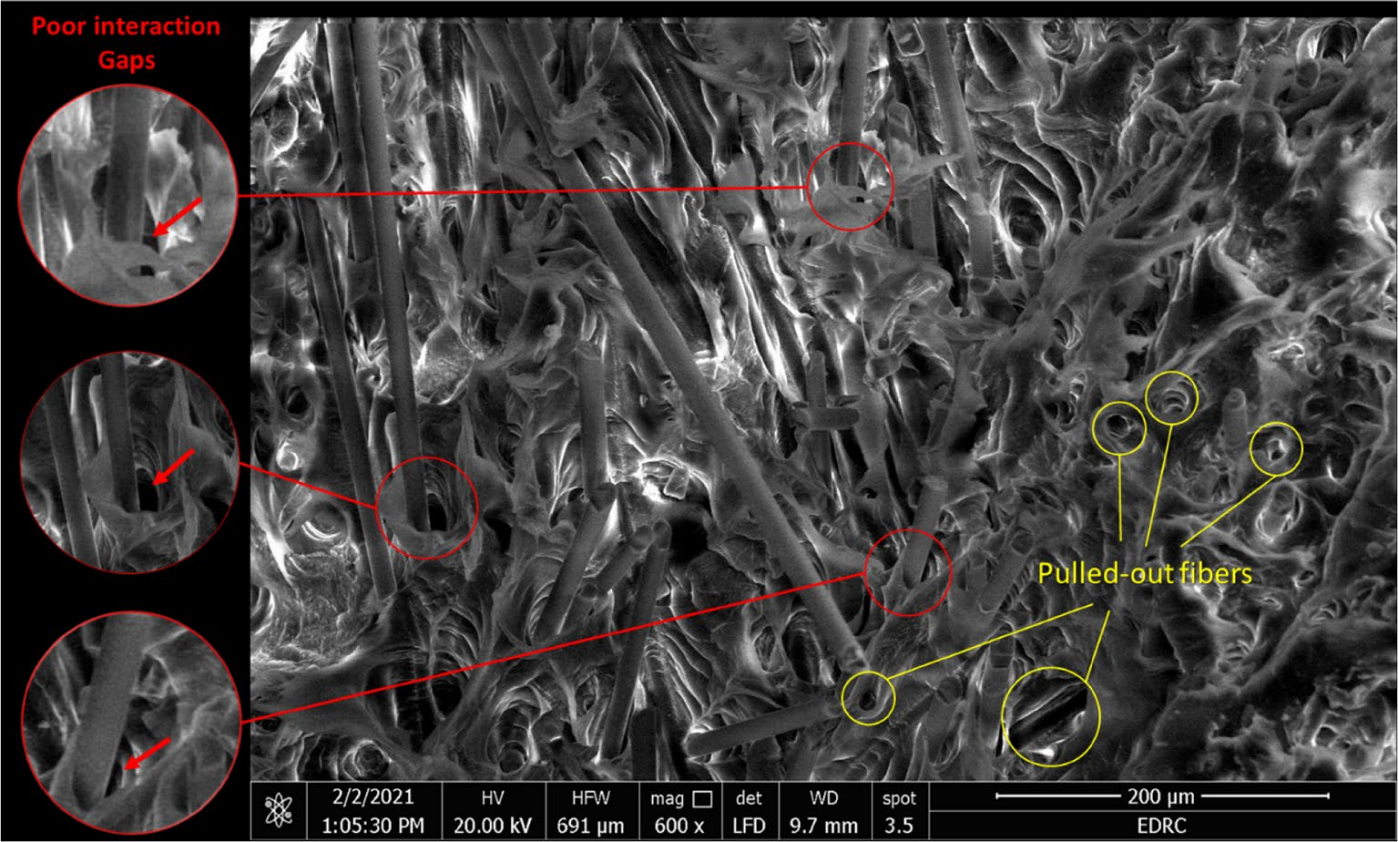
SEM of a fractured 30 wt% GFRPP specimen out of bearing strength test.

As could be noticed from Fig. 5, the percentage of the water sorption for all specimens is increasing as the specimens were immersed for longer periods. The water absorption seems to be increasing by introducing GF to PP and further increase occurs for higher fiber weight percentages. Interfaces between fibers and matrix induce capillary action penetrating the moisture through the composite which increases as more interfaces are present^44^. Also, gaps generated between fibers and matrix ascribing to a poor fiber-matrix interface in the absence of a coupling agent as explained by Mohebby et al.^18^, Valente et al.^45^, and Hernández‐Díaz et al.^46^. Mohebby et al.^18^ concluded that the addition of maleic anhydride-polypropylene to GF/Wood fibers reinforced PP hybrid composite decreases water absorption significantly^47^. Moreover, as Athijayamani et al.^48^ explained, the increased chance of microcracks existence in specimens containing more fiber content exemplifies increased water uptake on those composites. Also, the increased probability of impurities presence at higher fiber contents may increase the percentages of water absorption^28^, where PP and GF have hydrophobic natures^4^. Figure 6 represents scanning electron microscopy (SEM) of a fractured specimen illustrating the poor interaction gaps and fiber pullout in GFRPP composite. A jump in percentages of water absorption was observed for SFRPP specimens compared to neat PP and LFRPP specimens. SFRPP composites have a larger number of fibers than LFRPP composites for the same wt% as they have shorter fibers, where a larger amount of fibers is required to achieve the same wt%. Due to this fact, more fiber-matrix interactions are imposed with more generated gaps which may accept more moisture.

The calculated values of the Fickian diffusion coefficient, according to Eq. (2), for PP and GFRPP are shown in Table 4. The specimens with all compositions seemed to be following the Fickian diffusion where the rate of water sorption increased linearly as specimens were introduced to distilled water. Then, a nonlinear increase occurred as the specimens were kept for longer periods followed by a little increase approaching saturation points. The Fickian's diffusion could also be noticed in Fig. 5 for all types of specimens.

**Table 4:** Fickian diffusion coefficient values of PP and GFRPP with different fiber contents and lengths.

Specimen	Fickian diffusion coefficient (mm^2^/s)
PP	2.93E−06
WB1012	3.25E−06
WB2012	3.50E−06
WB2024	3.80E−06
WB3012	4.81E−06
WB1024	4.95E−06
WB3024	5.56E−06

### Bearing test results

#### Bearing strength

**Figure 7:**
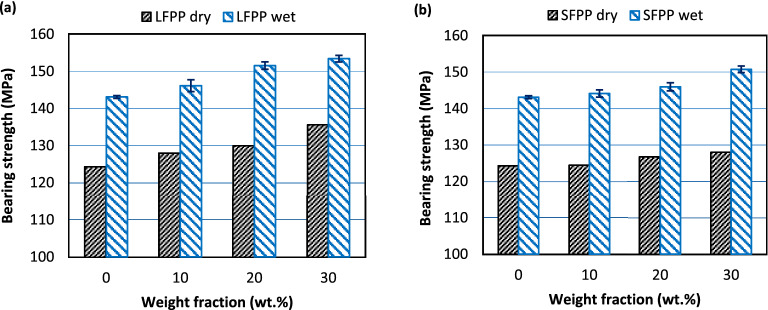
Bearing strengths in both dry and wet conditions for; (**a**) LFPP and (**b**) SFPP.

The bearing strengths obtained from the bearing tests in both dry and wet conditions were illustrated in Fig. 7. The addition of GF to PP increases the bearing strength of the material and as fiber wt% increases the bearing strength increases furthermore. An improvement of 9% could be noticed for DB3012 specimen above DB00 while only 3% improvement was obtained for DB3024 specimen above neat PP. Based on the obtained results the longer fibers, Fig. 7a, enhance the bearing strength more efficiently than short fibers, Fig. 7b. This result agrees with the results found in previous studies^33,34^. Subramanian et al.^33^ and Kumar et al.^34^ concluded that the strength of composite increases as the mean fiber length increases. Kumar et al.^34^ noticed that the strength of composite depends mostly on the fiber length over fiber content, and the reduced strength of the composite caused by decreased mean fiber length almost offsets the increased composite strength caused by higher fiber content. A similar result was obtained by Subramanian and Senthilvelan^49^ where the BS of leaf spring made from GFRPP was higher than that which made of pure PP and more increase in the bearing strength was obtained using GFRPP with longer fibers. Moreover, Asi^50^ showed that the bearing strength of glass fiber reinforced epoxy firstly increased as linear densities of woven fabric increased then decreased with an extra increase in woven fabric linear densities as a result of elevated void content and crimp levels of the obtained composite.

The same behavior was noticed for wet tested specimens where the bearing strength increases with increasing fiber wt%, also the longer fibers showed more enhancement in the bearing strength than shorter ones, Fig. 7b.

By comparing the bearing strengths of dry and wet tested specimens in Fig. 7, an obvious jump in the material bearing strengths in the wet condition is obtained as compared to dry ones applied for all types of specimens. This jump in the wet environment even exceeds the enhancements due to GF addition in the dry condition where the strength of WB00 is 5% and 12% higher than DB3012 and DB3024, respectively. This behavior may be attributed to the existence of an incompressible liquid (water) penetrated into the material which increases the material resistance to the bearing load in the compressive portion of the bearing load subjected by the inserted pin. Ghasemzadeh-Barvarz et al.^51^ showed an increase in the tensile strength and modulus of neat PP and hybrid flax-fibers/GF/PP composite after 42 days of immersion in distilled water. Also, Bergeret et al.^8^ noticed an increase in impact strength of PA66/GF composite at early times of aging. Hassan et al.^52^ observed an increment in the flexural strength of PA66/GF for wet specimens (50% RH) than the dry condition.

#### Bearing strain (strain at break)

**Figure 8:**
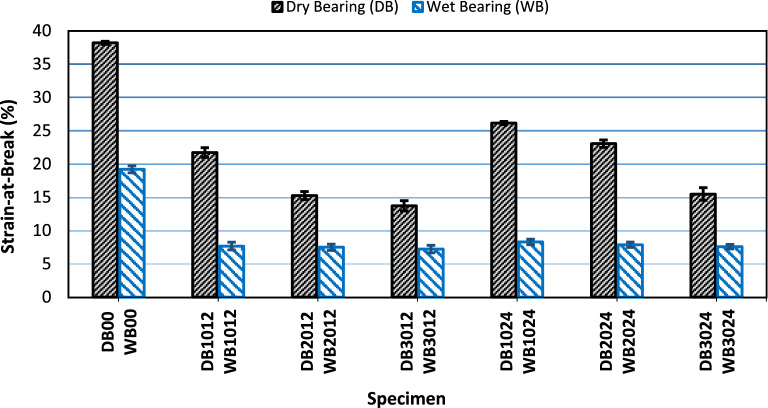
Strain-at-break for dry and wet specimens tested in bearing.

On the contrary, the strain-at-break between wet and dry conditions showed the opposite behavior. Where Fig. 8 represents the difference in the strain at break of the specimens tested in bearing between wet and dry conditions. It could be clearly observed from Fig. 8 that, the strain at break dramatically decreases when specimens are subjected to water immersion. The same behavior was observed for all specimens. The penetrated water fills the microcracks in the material which accelerates the crack propagation^28,53^. Abdelhaleem et al.^28^ noticed an accelerated crack propagation for wet specimens than dry ones when studying the fatigue behavior of PP and GFRPP composites with different GF content. Meng et al.^53^ found that water ingress occurs as an effect of capillary and the mass of water conserved during the loading cycle forbidding cracks from closing after load removal which in turn accelerates the crack propagation. The absorbed water chemically reacts with the GF in the composites. Chemical elements vanished as an effect of this reaction and were replaced with micro-flaws which are the seeds of fiber fractures^28^. Also, Ghasemzadeh-Barvarz et al.^51^ observed a decrease in both elongation-at-break and strain-at-yield of PP and GFRPP after being water aged for 1000 h.

### FTIR spectroscopy

**Figure 9:**
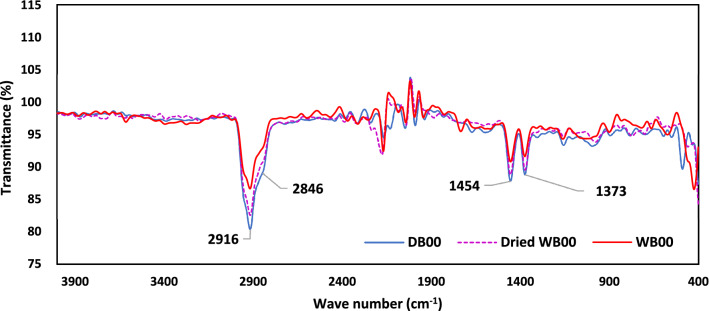
Result of FTIR test for PP specimen.

**Figure 10:**
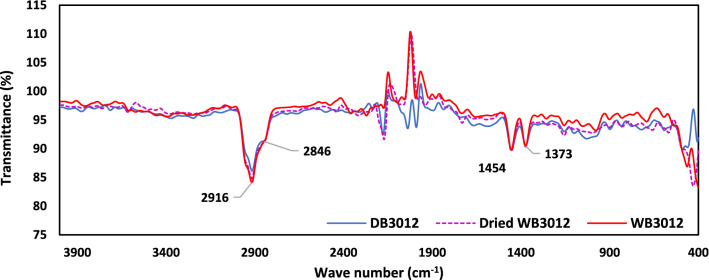
Result of FTIR test for GFRPP specimen.

Results of FTIR spectroscopy of PP and GFRPP specimens before and after water immersion are shown in Figs. 9 and 10, respectively. Two merged peaks of Methylene (–CH_2_) exist at 2916 and 2846 cm^−1^ with asymmetrical stretching and symmetrical stretching of hydrogen atoms respectively as could be noticed in the diagnostic region of PP and GFRPP^54,55^. However, two adjacent peaks exist at the fingerprint region, between 400 and 1500 cm^−1^, of Methyl (–CH_3_) at 1454 and 1373 cm^−1^ with asymmetrical bending and symmetrical bending respectively^54–58^. All four mentioned peaks appeared in PP and GFRPP under dry, wet, and dried conditions. A group of peaks appeared in the region 2000–2300 cm^–1^, It is known that the existence of those peaks is attributed to the stretching vibrations of triple carbon–carbon bonds (C≡C)^59^. For PP the peaks seemed to have higher marginal values in dry condition and lower values appeared for dried specimens while the lowest marginal value owed to wet specimens. It is likely that the absorption of water leads to a gradual decrease in the intensity of radiation passing through the sample^59^. Compared to neat PP, the peaks’ marginal values of dry, wet, and dried specimens in GFRPP are much closer to each other. The OH peaks which represent the exitance of moisture are not noticed in any sample. This may be occurred due to the removal of the surface moisture before the tests^60–63^.

The effect of water absorption on the chemical composition of PP or GFRPP specimens could not be clearly detected from the FTIR spectroscopy tests. The peaks that appeared in all specimens’ conditions (dry, wet, and dried) could not be specifically distinguished from one another and hence the change in the chemical composition due to water immersion for different specimens’ conditions was not occurred.

### Stress–strain curves

**Figure 11:**
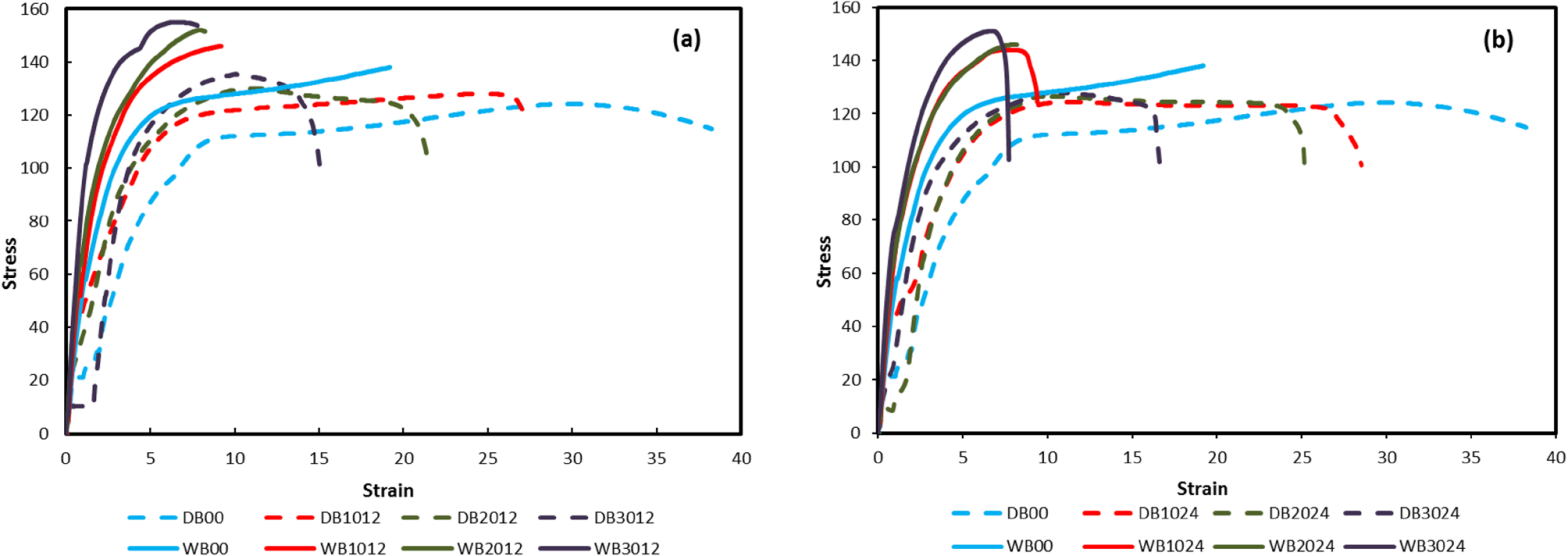
Stress–Strain curves of Polypropylene and Glass fiber reinforced Polypropylene in both dry and wet conditions for; (**a**) LFRPP and (**b**) SFRPP.

Stress–strain curves of PP and GFRPP specimens with different weight fractions and fiber lengths are illustrated in Fig. 11, for both dry and wet bearing tests. These curves accumulate the multiple effects of moisture on both bearing strength and bearing strain. As could be noticed, a recognizable increase in specimens’ strength for all wet-tested specimens over dry ones; accompanied by a drop in the materials’ strains. This behavior is definitely an indication of the different responses of the materials between dry and wet bearing tests.

### Analysis of specimen’s failure due to bearing test

**Figure 12:**
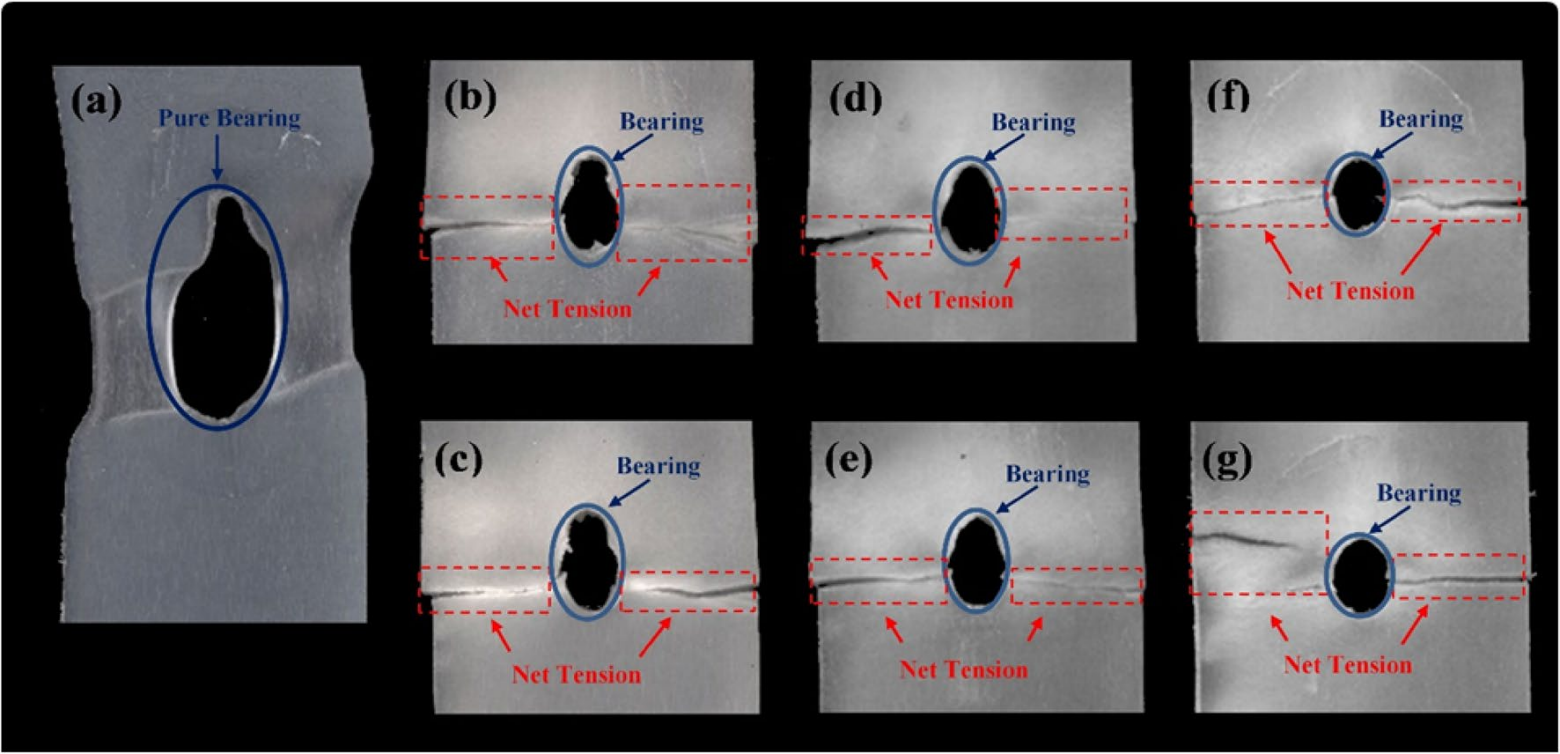
Failure morphologies of specimens tested in bearing in dry condition; (**a**) DB00, (**b**) DB1012, (**c**) DB1024, (**d**) DB2012, (**e**) DB2024, (**f**) DB3012, and (**g**) DB3024.

**Figure 13:**
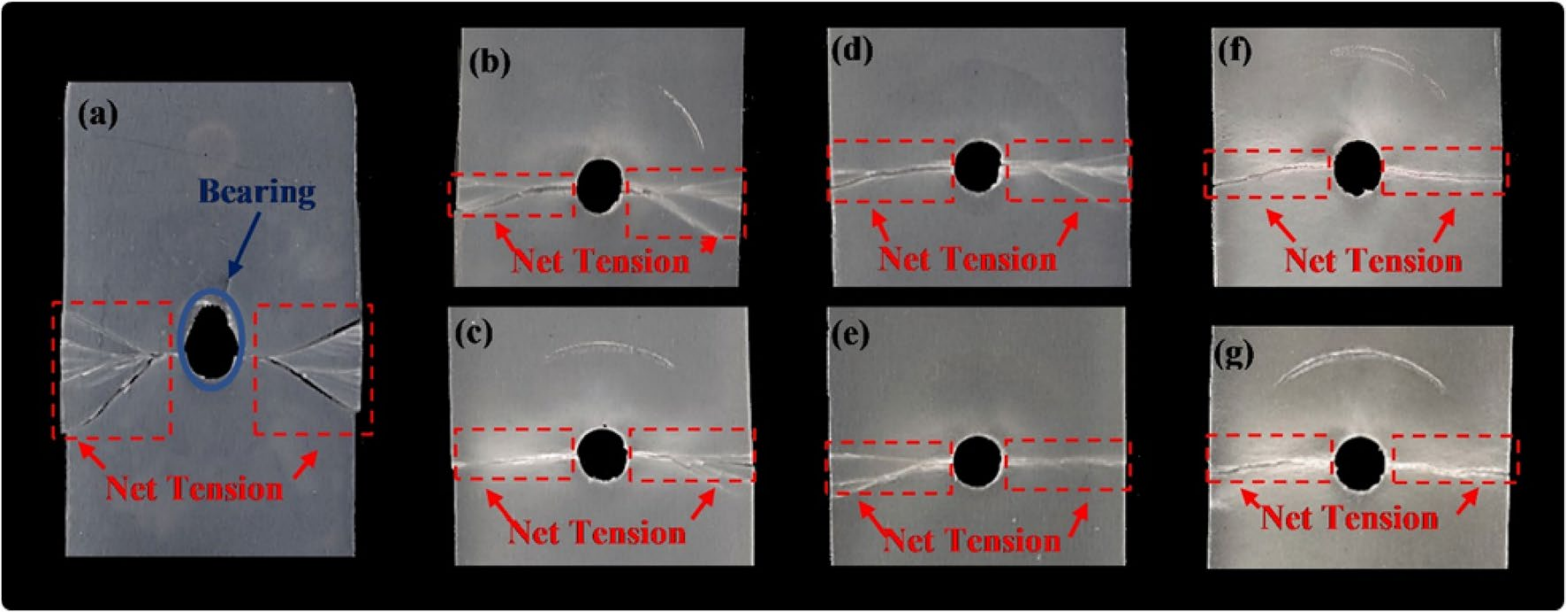
Failure morphologies of specimens tested in bearing in wet condition; (**a**) WB00, (**b**) WB1012, (**c**) WB1024, (**d**) WB2012, (**e**) WB2024, (**f**) WB3012, and (**g**) WB3024.

There are basically four failure modes of the materials under bearing load noticed out of bearing strength test; bearing, net-tension, shear out, and cleavage failure modes. The most ideal failure mode is bearing failure however other failure modes are considerably recommended for materials under bearing loads^20,64,65^. Damages that occurred to fiber-reinforced composites could be related to fracture of fibers, matrix cracking, fiber-matrix interfacial bonding failure, and/or their combinations^66^. Investigation of the failed surfaces of the composites in this study clarifies the failure modes, where the failure morphology out of dry and wet bearing tests are shown in Figs. 12 and 13, respectively.

A pure bearing failure mode was noticed for neat PP specimens (DB00), Fig. 12a, while a combined bearing and net-tension failure modes were observed for GFRPP composite specimens, Fig. 12b–g. Almost the same failure modes between different fiber lengths are noticed. A decrease in the bearing capacity was observed as the fiber wt% increased. Therefore, for DB3012 and DB3024, Fig. 12f,g, specimens bearing failure has barely occurred, while impressive bearing capacity is obtained by neat PP as indicated in Fig. 12a.

However, observed failure modes for wet tested specimens in Fig. 13 differ from those of dry specimens in Fig. 12. Wet neat PP specimens failed in mixed mode failure, Fig. 13a, while the wet GFRPP specimens failed almost under net-tension with a little bearing deformation, Fig. 13b–g. The change in the failure mode of neat PP specimens from pure bearing in dry conditions to mixed failure in wet conditions might be related to accelerated crack propagation and the presence of fiber flaws due to moisture, as explained in “Bearing test results”, that led to the reduction in the strain-at-break as shown in Fig. 8.

## Conclusions

The present work investigated the bearing strength at dry and wet conditions for PP and GFRPP composites with different GF weight fractions. The results revealed the following:

- Longer initial fibers in the injection molding process do not guarantee longer fibers in the produced composites. Specimens having longer FFSL (24 mm) have much smaller fibers in the resulting composites than specimens with smaller FFSL (12 mm).
- GFRPP composites absorb water more than neat PP as an effect of gaps presence at fiber-matrix interfaces and increased levels of contaminations. Moreover, more water was absorbed in specimens with higher contents of fibers due to more fiber-matrix interfaces at higher fiber weight fractions.
- A jump in water absorption was observed for SFRPP than LFRPP for the same fiber weight fraction, where more fibers exist with more fiber-matrix interfaces that accept more moisture at the same weight percentage.
- For both dry and wet bearing tests, the bearing strength increased with the increase in fiber weight fractions and for longer fiber length.
- The values of bearing strengths of wet tested specimens were higher than dry tested specimens, while strain-at-break had lower values for wet specimens.
- The results of FTIR spectroscopy test indicated that, the effect of water absorption on the chemical composition of PP or GFRPP specimens could not be clearly detected, and hence the change in the chemical composition due to water immersion for different specimens’ conditions was not occurred.
- The fracture morphologies of dry and wet specimens were not the same, where dry neat PP failed under pure bearing, and wet neat PP failed under mixed failure mode (net tension and bearing). The dry GFRPP specimens showed mixed-mode failure while wet GFRPP almost showed net tension failure.

## Data Availability

All data generated or analyzed during this study are included in this published article.
